# Systematic Reviews and Meta-Analyses of Traditional Chinese Medicine Must Search Chinese Databases to Reduce Language Bias

**DOI:** 10.1155/2013/812179

**Published:** 2013-10-08

**Authors:** Xin-Yin Wu, Jin-Ling Tang, Chen Mao, Jin-Qiu Yuan, Ying Qin, Vincent C. H. Chung

**Affiliations:** ^1^Division of Epidemiology, JC School of Public Health and Primary Care, The Chinese University of Hong Kong, 4/F, School of Public Health Building, Prince of Wales Hospital, Shatin, New Territories, Hong Kong; ^2^Shenzhen Municipal Key Laboratory for Health Risk Analysis, Shenzhen Research Institute, The Chinese University of Hong Kong, 10 Yuexing Erdao, Nanshan District, Shenzhen, Guangdong Province, China; ^3^The Hong Kong Branch of the Chinese Cochrane Centre, Faculty of Medicine, The Chinese University of Hong Kong, Shatin, New Territories, Hong Kong

## Abstract

Systematic reviews (SRs) that fail to search non-English databases may miss relevant studies and cause selection bias. The bias may be particularly severe in SRs of traditional Chinese medicine (TCM) as most randomized controlled trials (RCT) in TCM are published and accessible only in Chinese. In this study we investigated how often Chinese databases were not searched in SRs of TCM, how many trials were missed, and whether a bias may occur if Chinese databases were not searched. We searched 5 databases in English and 3 in Chinese for RCTs of Chinese herbal medicine for coronary artery disease and found that 96.64% (115/119) eligible studies could be identified only from Chinese databases. In a random sample of 80 Cochrane reviews on TCM, we found that Chinese databases were only searched in 43 or 53.75%, in which almost all the included studies were identified from Chinese databases. We also compared SRs of the same topic and found that they may draw a different conclusion if Chinese databases were not searched. In conclusion, an overwhelmingly high percentage of eligible trials on TCM could only be identified in Chinese databases. Reviewers in TCM are suggested to search Chinese databases to reduce potential selection bias.

## 1. Introduction

Systematic reviews (SRs) provide authoritative, summary of evidence for informing medical decisions. A good SR should identify and include all studies regardless their place of conduct and year and language of publication that meet pre-defined eligibility criteria so as to reduce selection biases [1]. A study showed that only half of all published clinical trials could be identified if only MEDLINE was searched [2]. Searching at least two electronic databases was considered essential for assuring the validity of SRs [3].

Selection bias is a major threat to the validity of SRs and often caused by language restriction in literature search as many review teams find it difficult to access non-English language databases and subsequently fail to include relevant studies. It is known that trials with statistical significant results are more likely to be published in English [4], and thus a SR that includes studies published only in English would overestimate the true effect, although in some areas an opposite bias may be true.

An analysis [5] of 159 SRs which included a total of 600 clinical trials found that the proportion of non-English studies varied largely depending upon the topic or area of interest. For instance, in rheumatology and orthopaedics 35.71% of trials were published in languages other than English, whereas this number was only 10.14% in tobacco addiction and 20.34% in vision research [2, 5].

As many trials in traditional Chinese medicine (TCM) may be published only in Chinese and available in Chinese databases, a failure to search databases in Chinese may miss many relevant studies and cause biases in SRs. How often have SRs of TCM failed to search Chinese databases? How many trials could be missed if Chinese databases are not searched? How would the missed trials affect the overall combined effect of a SR? We conducted this study to shed light on these questions.

## 2. Materials and Methods

### 2.1. Comparing the Number of Randomized Controlled Trials (RCTs) on a TCM Topic Identified in International and Chinese Databases

 This study has three components. In this part, we estimated and compared the number of RCTs of Chinese herbal medicine (CHM) for treating coronary artery disease (CAD) identified from international databases with that identified in databases in Chinese.

Five major international databases (MEDLINE, EMBASE, CINAHL, CENTRAL, and AMED) and 3 major Chinese databases (Chinese Biomedical Database, Chinese Medical Current Contents and Taiwan Periodical Literature Database) were searched from their inception to July 2010 to identify RCTs of CHM for treating CAD. Regarding CHM, the search included general terms and phrases for TCM and CHM and specific names of propriety Chinese herbal medicines and individual herbs that are commonly used for treating CAD. We compiled an exhaustive list of relevant terms and phrases by carefully studying relevant texts such as systematic reviews, narrative reviews, RCTS, and textbooks and by consulting TCM practitioners in the relevant field. We also used highly sensitive search strategies for RCTs and for CAD and finally limited to human studies only. Our detailed search strategies were attached in Appendix 1 In the Supplementary Material available online at http://dx.doi.org/10.1155/2013/812179.

A study was considered eligible if (1) it is a RCT in study design, (2) it used CHM as the tested treatment compared with no treatment or a placebo treatment and often as an add-on treatment on top of routine western medicine treatment given to patients in all comparison groups, and (3) participants are adults of any age or ethnic origin with a CAD including angina and myocardial infarction. CHM is defined as any Chinese herbal preparations that contain at least one herb that is included in the latest version of the Chinese Pharmacopeia [6]. The duration of treatment and observation of the RCT must be 7 days or longer. Primary outcomes of concern were deaths from acute myocardial infarction (AMI) or any other causes. Secondary outcomes included recurrent AMI, stroke, angina, revascularization, and quality of life. RCTs identified were estimated and reported separately for international databases and Chinese databases. Studies that can be identified from both types of databases were not common and considered as “identified from international databases.” Details of the search strategy and inclusion criteria have been published elsewhere [7].

 Finally, we estimated and compared the overall efficacy of CHM in reducing the risk of angina in patients with unstable angina as there were trials available in both international and Chinese databases. A funnel plot was drawn to show how studies identified in international databases may differ from those in Chinese databases.

### 2.2. Comparing the Number of RCTs in 2 SRs of the Same Topic with and without Search of Chinese Databases

In this part, we identified and compared 2 independent SRs of RTCs that addressed the same clinical question, but one searched Chinese databases and the other did not. By “the same clinical question,” it means that the RCTs have the same PICO or are common in the test TCM therapy, patients, control treatment, and clinical outcomes. We compared the two reviews in the number of RCTs identified by sources, combined results, and conclusions to see how likely the result would differ if Chinese databases were not searched.

### 2.3. Percentage of Cochrane SRs That Searched Chinese Databases

We randomly selected 80 systematic reviews on TCM from the Cochrane Database of Systematic Reviews (CDSR) and investigated how often Chinese databases were searched in systematic reviews of TCM and the ratio of RCTs identified between international and Chinese databases.

 We searched the January Issue of the 2013 CDSR for SRs on TCM by using Chinese medicine, Chinese herbal medicine; and acupuncture. Eighty SRs were sampled at random from 625 Cochrane SRs which are likely to include trials that have at least one arm of TCM therapy such as herbal medicine, acupuncture, and other forms of TCM. Detailed search strategies are attached in Appendix 2. If a study can be found in international databases, it will be considered “identified from international databases” regardless whether or not it can be found in Chinese databases. 

We estimated the number and percentage of (1) SRs that searched both international and Chinese databases, (2) SRs that included a Chinese author, (3) RCTs that were identified from Chinese databases, and (4) SRs that tended to support for an efficacy of TCM treatment. 

Statistical Package for the Social Sciences 18.0 (SPSS18.0), STATA 11 and Review Manager 5 were used for statistical analyses.

## 3. Results

### 3.1. RCTs of CHM in Treating CAD Identified in International and Chinese Databases

**Figure 1:**
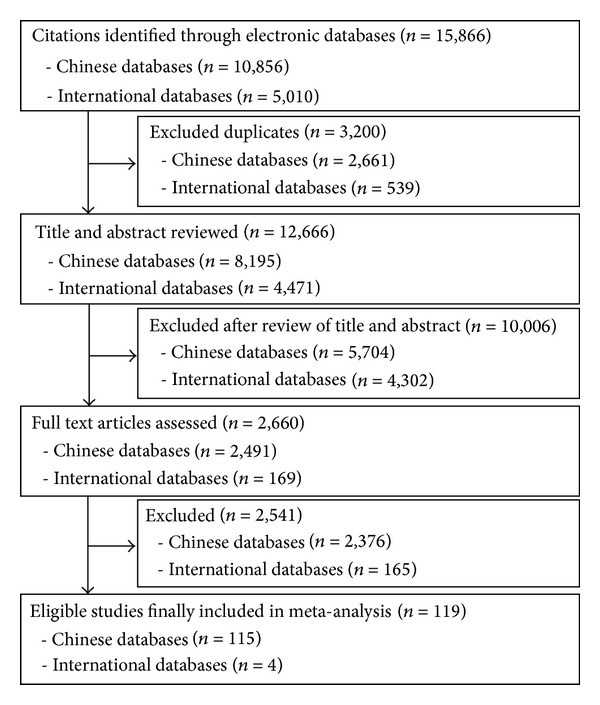
Flow chart of literature search and study selection for systematic reviews of Chinese herbal medicine for treatment of coronary artery disease.

Fifteen thousand eight hundred and sixty-six citations were retrieved from the electronic databases searched, with 10,856 from Chinese databases and 5,010 from international databases. After exclusion of duplicates, 12,666 citations were retained for further screening. Another 10,006 were considered irrelevant and excluded purely according to the title and abstract. Scrutiny of the full texts was conducted for the remaining 2,660 citations. At last, a total of 119 papers met the eligibility criteria and were included in this analysis. Details of the literature search and studies identified in each step are shown in Figure 1. 

96.64% (115/119, 95% confidence interval (CI) = 93.41% ~ 99.87%) of the RCTs were published in Chinese and available only in Chinese databases, with only 3.36% (4/119, 95% CI = 0.13%~6.59%) available in international databases. RCTs that were both available in international and Chinese databases were not found. 

Twelve eligible trials were found that used CHM to treat angina patients for preventing further attacks of angina, with 1 identified from international databases [10] and 11 from Chinese databases [11–21]. The RCT identified from international databases showed a relative risk (RR) of 1.04 with 95% CI = 0.07~16.18, suggesting the treatment be ineffective. However, combining all the 12 trials resulted in an RR of 0.36 with a 95% CI = 0.26~0.51, suggesting the treatment can reduce the risk of angina by 64%. In addition, by using the Cochrane Risk of Bias Tool [1], we assessed the methodological quality of the 12 trials and did not find any major difference in bias-prevention methods used in the trials identified from Chinese databases from that identified in English databases except allocation concealment, which could well be a problem of report in particular in some early trials rather than conduct.

**Figure 2:**
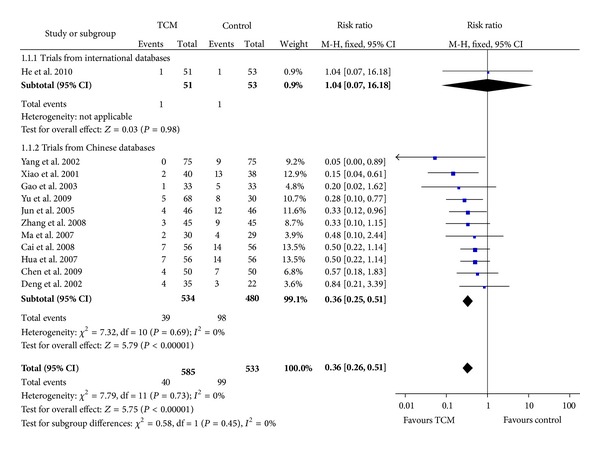
Meta-analysis of trials of traditional Chinese medicine for treatment of unstable angina to prevent reattack of angina, according to databases from which RCTs were identified.

**Figure 3:**
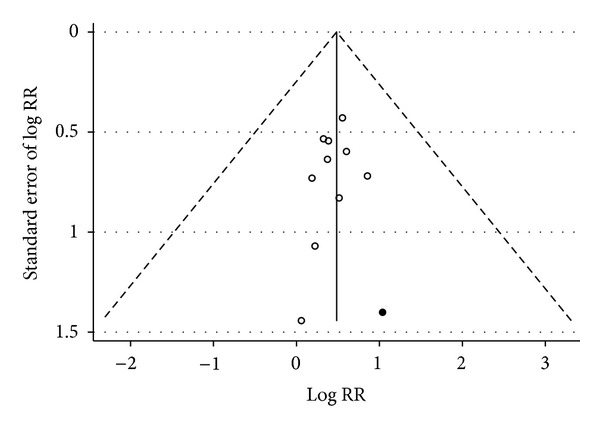
Funnel plot of RCTs of traditional Chinese medicine for treatment of unstable angina according to databases from which the studies were identified (the solid dot represents the trial identified from international databases, and the black dots represent trials from Chinese databases).

 Details of the results are shown in Figure 2. Egger's test showed no evidence for the presence of publication bias among the 12 studies (*t* = −0.33, *P* = 0.75). The trial identified in international databases showed the smallest result (Figure 3).

### 3.2. Comparison of Two SRs on Acupuncture for Chronic Asthma

Two independent SRs [8, 9] of acupuncture for chronic asthma were identified and compared. Both SRs reviewed RCTs on the efficacy of acupuncture for treating chronic asthma as compared with any other treatment rather than acupuncture and using as outcomes peak expiratory flow rates (PERF), forced vital capacity (FVC), and forced expiratory volume in one second (FEV1). McCarney and colleagues [8] did not search Chinese databases and did not set time restrictions and found 12 trials, whereas Yu and colleagues [9] searched both English and Chinese databases for trials published between January 2000 and October 2009 and found 22 trials with 17 of them published in Chinese. Only 1 trial was included in both SRs.

The review by McCarney and colleagues [8] did not reach a conclusion about the efficacy of acupuncture for treatment of chronic asthma, whereas Yu and colleagues [9] concluded that acupuncture could significantly improve the overall improvement of asthma patients. Detailed comparisons of the two SRs were shown in Table 1.

### 3.3. Percentage of Cochrane SRs That Searched Chinese Database

Of the 80 SRs of TCM trials randomly selected from the CDSR, 43 (53.75% with 95% CI = 42.82%~64.68%) searched Chinese databases and 37 (46.25%) did not. Of the 43 SRs that search Chinese database, 11 (25.58%) were on acupuncture, 2 (4.65%) were on massage, and 30 (69.77%) were on herbal medicine or other forms of TCM. Of the 37 SRs that did not searched Chinese databases, 6 (16.22%) were on acupuncture, 4 (10.81%) on massage and/or aromatherapy, and 27 (72.97%) on herbal medicine or other forms of TCM. No statistical difference was found in the types of treatment between SRs that searched Chinese database and those that did not (*χ* ^2^ = 1.856, *P* > 0.05). 

The average number of RCTs included was similar in the SRs that searched Chinese databases and those that did not. Almost all (*P*_25_ ~ *P*_75_ = 87.85% ~ 100.00%) the RCTs in the 43 SRs that searched Chinese databases were identified from the Chinese databases, and as expected no RCT was identified from Chinese databases in the 37 SRs that did not search Chinese databases. 15 (34.88%) SRs that searched Chinese database and 12 (32.43%) SRs that did not search Chinese database drew a conclusion that tended to support for an efficacy of TCM. In addition, 41 of the 43 SRs (95.35%, 95% CI = 89.06%~100.00%) that searched Chinese databases had included Chinese coauthors, while only 4 (10.81%) of the 37 SRs that did not search Chinese databases had Chinese coauthors. Details are shown in Table 2.

## 4. Discussion

Comprehensive literature search is essential for a systematic review [1, 3, 22]. Our SRs of TCM for treating CAD found that 96.64% of the eligible RCTs were identified from Chinese databases. A review of 80 SRs randomly selected from the CDSR showed that a similar percentage of eligible studies were identified from Chinese databases if both international and Chinese databases were searched, but only 53.75% of the SRs searched Chinese databases. We also demonstrated with examples that major disagreements in conclusion might occur if Chinese databases were not searched, although it remains uncertain how the differences should be accounted for. 

Language restriction is very common in systematic reviews although to various degrees. A review [23] found that among 79 meta-analyses, only 19 (24.05%) included non-English RCTs, while another study of 130 meta-analyses [24] reported a higher percentage (48/130 = 36.92%). Gregoire and colleagues [25] reported that 77.78% (28/36) restricted language of publications, with the majority of the meta-analysis (92.86%) included only RCTs published in English. 

It is uncertain whether failure to search non-English databases would affect the overall result and conclusion. For example, a review [23] of 79 meta-analyses found that meta-analyses with and without language restriction did not differ in the estimate of the overall effect. In contrast, a review [25] of 36 meta-analyses found that exclusion of trials for linguistic reasons produced different results from meta-analyses without linguistic restrictions. 

It is very likely that the language bias is dependent on the topic of the review and that the direction of bias is unpredictable. For example, a review [26] of 42 systematic reviews found that language restriction did not affect the results of SRs in conventional medicine but tended to lead to underestimation of the results of SRs in complementary and alternative medicine. It is also shown that the estimate of effect was greater in non-English language trials than in English language trials [5].

 In addition, failure to include relevant studies in languages rather in English may not only bias the result but also reduce the statistical precision of the overall estimate and reduce the possibility of meaningful subgroup analyses which may result in important findings on factors that may affect the efficacy of treatment. 

 Some may argue that trials published in languages other than English was of methodological quality lower than those in English and therefore reviews do not need to include these low quality trials. This is a relevant argument against search of non-English databases but may not generally hold. For example, a review [4] of 40 pairs of RCTs matched in the first author and year of publication found that the methodological quality measured by a methodological quality scale was comparable between those published in English and those in German, although trials in English were more likely to report a statistically significant result than trials in German. Similarly, a study [27] that compared the methodological quality on randomization of 8 trials from English databases with 28 trials from Chinese databases on TCM for chronic fatigue syndrome found that trials from different sources were similar in quality, but searching Chinese databases increased greatly the number of potentially relevant articles. As shown above, a similar result is also observed in our study. 

 These findings suggest that relevant trials published in different languages should all be included in a systematic review. As discussed above, the relative quality of Chinese trials and English trials is not precisely predictable, although often trials in Chinese may have a lower quality than those in English. There is no good reason to believe that studies published in non-English languages are always of low quality. Thus, studies which are deemed low quality should be excluded only after they have been identified and assessed in their quality. 

Our study also showed that 89.19% of the SRs that did not search Chinese databases did not have Chinese coauthors, suggesting that lack of Chinese collaborators is probably one of the major reasons why Chinese databases had not been searched in many SRs in TCM. Given the high percentage of trials which can be found by searching Chinese databases, an organized approach to searching Chinese databases would be highly desirable for those who wish to conduct SRs in TCM. Before such a service is created, we suggest reviewers for identifying Chinese colleagues to collaborate on TCM reviews, possibly through the contact of the Chinese Cochrane Centre, Hong Kong Cochrane Branch, and other active groups in conducting TCM reviews in China.

## 5. Conclusion

In conclusion, an overwhelmingly high percentage of eligible trials on TCM can be identified only through searching Chinese databases. However, some 50% of systematic reviews in TCM failed to search Chinese databases, which may lead to a bias of substantive degree and of unpredictable direction. In order to reduce this language bias, researchers are suggested to include Chinese speaking colleagues as collaborators from the very beginning if they wish to conduct reviews in TCM.

## Supplementary Material

Appendix1, searching strategies of CHM for CAD: Five major international databases (MEDLINE, EMBASE, CINAHL, CENTRAL and AMED) and 3 major Chinese databases (Chinese Biomedical Database, Chinese Medical Current Contents, and Taiwan Periodical Literature Database) were searched from their inception to July 2010 to identify RCTs of CHM for treating CAD. Regarding CHM, the search included general terms and phrases for TCM and CHM and specific names of propriety Chinese herbal medicines and individual herbs that are commonly used for treating CAD.
Appendix2 Searching strategy of systematic reviews on traditional Chinese medicine in the Cochrane Database of Systematic Reviews (CDSR): This appendix shows the details and results of our search for systematic reviews on Chinese herbal medicine and acupuncture in the 2013 January Issue of the Cochrane Database of Systematic Reviews.

## Figures and Tables

**Table 1:** Comparison of 2 systematic reviews on acupuncture for chronic asthma.

	McCarney et al., 2004 [8]	Yu et al., 2010 [9]
Journal	Cochrane Database of Systematic Reviews	Chinese Acupuncture and Moxibustion
Time period for included studies	Till August 2008	January 2000–October 2009
Chinese databases searched	0	3
International databases searched	3	2
Number of RCTs identified	12	22
Number and % of RCTs identified from Chinese databases	0 (0.00%)	17 (77.27%)
Total number of patients	350	3058
Publication bias	Unable to assess due to a limited number of included studies on each outcome	No clear publication bias for outcomes including total improvement rate, FEV1, and PEFR, unable to assess publication bias on FVC due to a small number of included studies
Main results	No statistically significant or clinically relevant effects were found for acupuncture when compared to sham acupuncture	The acupuncture group showed a greater total improvement rate and significantly improved PEFR, FVC, and FEV1/FVC as compared to its control
Conclusion	No enough evidence for making any conclusions on the value of acupuncture in the treatment of asthma	Acupuncture can significantly improve the overall improvement, PEFR, FVC, and FEV1/FVC

*PEFR: peak expiratory flow rates; FVC: forced vital capacity; FEV1: forced expiratory volume in one second.

**Table 2:** Comparison of SRs in TCM that searched Chinese databases with those that did not.

	Whether Chinese databases were searched		*P* value for differences
	Yes	No	
Number of SRs	43	37	
SRs that included Chinese authors	41 (95.35%)	4 (10.81%)	<0.001
Median number of included studies (range)	8.00 (0–75)	6.00 (0–36)	0.58
Median number of studies from Chinese databases (range)	8 (0–74)	0	<0.001
Number (%) of SRs tended to support for an efficacy of TCM treatment	15 (34.88%)	12 (32.43%)	0.82
% of studies from Chinese databases (range)	100.00% (0.00–100.00%)	0	<0.001
