# Supplementary material for: Systematic Reviews and Meta-Analyses of Traditional Chinese Medicine Must Search Chinese Databases to Reduce Language Bias
Source: Evid Based Complement Alternat Med. 2013 Oct 8;2013:812179. doi: 10.1155/2013/812179 (PMC3816048; doi:10.1155/2013/812179)
Supplement: Supplementary file 1 — Appendix1, searching strategies of CHM for CAD: Five major international databases (MEDLINE, EMBASE, CINAHL, CENTRAL and AMED) and 3 major Chinese databases (Chinese Biomedical Database, Chinese Medical Current Contents, and Taiwan Periodical Literature Database) were searched from their inception to July 2010 to identify RCTs of CHM for treating CAD. Regarding CHM, the search included general terms and phrases for TCM and CHM and specific names of propriety Chinese herbal medicines and individual herbs that are commonly used for treating CAD. Appendix2 Searching strategy of systematic reviews on traditional Chinese medicine in the Cochrane Database of Systematic Reviews (CDSR): This appendix shows the details and results of our search for systematic reviews on Chinese herbal medicine and acupuncture in the 2013 January Issue of the Cochrane Database of Systematic Reviews. [file 812179.f1.pdf]

Appedix1, searching strategies of CHM for CAD

1: MEDLINE: Search strategy and results

1 Clinical trial.mp. (513559)
2 clinical trial.pt. (462927)
3 random:.mp. (637094)
4 tu.xs. (2916488)
5 randomized controlled trial.pt. (293863)
6 controlled clinical trial.pt. (81845)
7 placebo.ab. (120044)
8 drug therapy.fs. (1391575)
9 trial.ab. (207828)
10 trial.mp. (672924)
11 trial$.mp. (908556)
12 groups.ab. (978640)
13 group$.mp. (2004834)
14 1 or 2 or 3 or 4 or 5 or 6 or 7 or 8 or 9 or 10 or 11 or 12 or 13 (5019226)
15 exp Myocardial Ischemia/ (309698)
16 myocard$ ischemi$.mp. (40327)
17 exp Heart Diseases/ (754872)
18 heart disease$.mp. (151767)
19 angina.mp. (53904)
20 (heart adj3 disease$).mp. (167534)
21 (coronary adj3 disease$).mp. (178631)
22 myocardial infarct$.mp. (158996)
23 exp Myocardial Revascularization/ (67471)
24 myocardi$ revascular$.mp. (10317)
25 (coronary adj3 bypass$).mp. (46614)
26 cabg.mp. (9383)
27 (coronary adj3 angioplast$).mp. (31563)
28 ptca.mp. (6025)
29 (heart adj3 infarct$).mp. (5615)
30 postmyocardial infarct$.mp. (730)
31 15 or 16 or 17 or 18 or 19 or 20 or 21 or 22 or 23 or 24 or 25 or 26 or 27 or 28 or 29 or 30 (826923)
32 exp Drugs, Chinese Herbal/ (19792)
33 Chinese herb$.mp. (20924)
34 exp Medicine, Chinese traditional/ (8556)
35 Traditional Chinese medic$.mp. (4943)
36 exp Phytotherapy/ (21333)
37 phytother$.mp. (21442)
38 (chinese adj5 (traditional or medicine$)).mp. (14635)
39 (herbs or herbal).mp. (30662)

40 (plant or plants).mp. (364139)

41 (traditional adj5 medicine$).mp. (23836)

42 (She Xiang Bao Xin or Su Xiao Jiu Xin or Fu Fang Dan Shen or Guan Xin Dan Shen or Su Bing or Su Xin or Tong Xin Luo or Hu Xin or Jiu Xin or Huan Xin or She Xiang Su He or She Xiang or Zhu She Xiao Shuan or Huo Xin Dan or Yi Xin Wan or Xin Ling Wan or She Xiang Xin Nao Le or Kuan Xiong or Xin Tong or Fu Fang Xi Xin or Guan Xin).mp. (22)

43 (Xin Jiao Tong Ning or Jiu Xin or Ge Gen Su or Shen Fu or Di Long or Hong Hua or Shu Xue Ning or Chuan Qiong Chin or Shu Xue Tong or Mai Pu Lin or Deng Zhan Xi Xin or Huang Qi or Ci Wu Jia or Guan Xin Ning or Tong Xin Luo or Nao Xin Tong or Bu Xin qi or Shen Shao or Nuo Di Kang or Yang Xin Shi or Xue Fu Zhu Yu or Xin Ke Shu).mp. (110)

44 (Di Ao Xin Xue Kang or Xin Yuan or Wen Xin or Sheng Mai or Yi Xin Shu or Xin Nao Shu Tong or Yin Xing Ye or Huo Xue Tong Mai or Xin Ke Ning or Xue Sai Tong or Xin Tong or Dan Lou or Xin Bao or You Gui or Xin Mai Tong or Tong Guan or Shen Mai or Deng Zhan Hua).mp. (51)

45 32 or 33 or 34 or 35 or 36 or 37 or 38 or 39 or 40 or 41 or 42 or 43 or 44 (399403)

46 14 and 31 and 45 (3609)

47 limit 46 to humans (2680)

48 limit 47 to ("article reviews (acp journal club)" or "article reviews (dare)" or evidence based medicine reviews or "topic reviews (cochrane)") (46)

49 limit 47 to "review" (579)

50 48 or 49 (603)

51 47 not 50 (2077)

52 from 51 keep 1-2077 (2077)

2: CENTRAL: Search strategy and results

1 exp Myocardial Ischemia/ (16340)
2 exp Heart Diseases/ (26917)
3 heart disease$.mp. (6541)
4 Myocardi$ Ischem$.mp. (2379)
5 exp Myocardial Revascularization/ (6464)
6 Myocardi$ Revascular$.mp. (817)
7 angina.mp. (6875)
8 (heart adj3 disease$).mp. (7160)
9 (coronary adj3 disease).mp. (10808)
10 (myocardial adj1 infarct$).mp. (11216)
11 (coronary adj3 bypass$).mp. (5512)
12 exp Coronary Artery Bypass/ (3887)
13 (coronary adj3 angioplast$).mp. (3186)
14 exp Angioplasty, Transluminal, Percutaneous Coronary/ (2515)
15 (heart adj3 infarct$).mp. (1429)
16 postmyocardial.mp. (90)
17 1 or 2 or 3 or 4 or 5 or 6 or 7 or 8 or 9 or 10 or 11 or 12 or 13 or 14 or 15 or 16 (38693)
18 exp Drugs, Chinese Herbal/ (1664)
19 chinese herb$.mp. (1943)
20 exp Medicine, Chinese Traditional/ (468)
21 traditional chinese med$.mp. (605)
22 exp Medicine, East Asian Traditional/ (508)
23 (chinese adj (traditional or medicine$)).mp. (1363)
24 (herbs or herbal).mp. (2721)
25 (plant or plants).mp. (4837)
26 exp Phytotherapy/ (2172)
27 (traditional adj3 medicine$).mp. (1439)
28 (She Xiang Bao Xin or Su Xiao Jiu Xin or Fu Fang Dan Shen or Guan Xin Dan Shen or Su Bing or Su Xin or Tong Xin Luo or Hu Xin or Jiu Xin or Huan Xin or She Xiang Su He or She Xiang or Zhu She Xiao Shuan or Huo Xin Dan or Yi Xin Wan or Xin Ling Wan or She Xiang Xin Nao Le or Kuan Xiong or Xin Tong or Fu Fang Xi Xin or Guan Xin).mp. (22)
29 (Xin Jiao Tong Ning or Jiu Xin or Ge Gen Su or Shen Fu or Di Long or Hong Hua or Shu Xue Ning or Chuan Qiong Chin or Shu Xue Tong or Mai Pu Lin or Deng Zhan Xi Xin or Huang Qi or Ci Wu Jia or Guan Xin Ning or Tong Xin Luo or Nao Xin Tong or Bu Xin qi or Shen Shao or Nuo Di Kang or Yang Xin Shi or Xue Fu Zhu Yu or Xin Ke Shu).mp. (46)
30 (Di Ao Xin Xue Kang or Xin Yuan or Wen Xin or Sheng Mai or Yi Xin Shu or Xin Nao Shu Tong or Yin Xing Ye or Huo Xue Tong Mai or Xin Ke Ning or Xue Sai Tong or Xin Tong or Dan Lou or Xin Bao or You Gui or Xin Mai Tong or Tong Guan or Shen Mai or Deng Zhan Hua).mp. (39)

31 18 or 19 or 20 or 21 or 22 or 23 or 24 or 25 or 26 or 27 or 28 or 29 or 30 (8619)
32 17 and 31 (479)

3: EMBASE: Complete search strategy and results

1 random$.mp. (493021)
2 factorial$.mp. (18460)
3 (crossover$ or cross-over$).mp. (47919)
4 placebo$.mp. (198663)
5 (doubl$ adj blind$).mp. (114351)
6 (singl$ adj blind$).mp. (14041)
7 assign$.mp. (122600)
8 allocat$.mp. (45111)
9 volunteer$.mp. (112947)
10 Crossover Procedure.sh. (23240)
11 Double-blind Procedure.sh. (78562)
12 Randomized Controlled Trial.sh. (191367)
13 Single-blind Procedure.sh. (9619)
14 random:.tw. (446443)
15 clinical trial:.mp. (677850)
16 exp health care quality/ (926899)
17 (random$ or placebo$).ti,ab. (497960)
18 ((single$ or double$ or triple$ or treble$) and (blind$ or mask$)).ti,ab. (108350)
19 controlled clinical trial$.ti,ab. (11693)
20 RETRACTED ARTICLE/ (3431)
21 Clinical trial/ (605247)
22 Randomized controlled trial/ (191367)
23 Randomization/ (28492)
24 Single blind procedure/ (9619)
25 Double blind procedure/ (78562)
26 Crossover procedure/ (23240)
27 Placebo/ (144300)
28 Randomi?ed controlled trial$.tw. (41070)
29 Rct.tw. (3639)
30 Random allocation.tw. (700)
31 Randomly allocated.tw. (11275)
32 Allocated randomly.tw. (1420)
33 (allocated adj2 random).tw. (575)
34 Single blind$.tw. (8228)
35 Double blind$.tw. (91015)
36 (triple adj blind$).tw. (153)
37 Placebo$.tw. (120147)
38 Prospective study/ (97004)
39 1 or 2 or 3 or 4 or 5 or 6 or 7 or 8 or 9 or 10 or 11 or 12 or 13 or 14 or 15 or 16 or 17 or 18 or 19 or 20 or 21 or 22 or 23 or 24 or 25 or 26 or 27 or 28 or 29 or 30 or 31 or 32 or 33 or 34 or 35 or 36 or 37 or 38 (1928006)
40 Case study/ (7285)

41 Case report.tw. (133625)
42 Abstract report/ or letter/ (543440)
43 40 or 41 or 42 (681573)
44 39 not 43 (1825352)
45 exp Ischemic heart disease/ (256257)
46 exp Heart Diseases/ (656662)
47 angina.mp. (48943)
48 (heart adj3 disease$).mp. (149300)
49 (coronary adj3 disease$).mp. (106491)
50 myocardial infarct$.mp. (93824)
51 exp Myocardial Revascularization/ (13215)
52 (coronary adj3 bypass$).mp. (43232)
53 cabg.mp. (8680)
54 (coronary adj3 angioplast$).mp. (21264)
55 ptca.mp. (5670)
56 heart infarct$.mp. (132549)
57 postmyocardial infarct$.mp. (616)
58 45 or 46 or 47 or 48 or 49 or 50 or 51 or 52 or 53 or 54 or 55 or 56 or 57 (728538)
59 exp Chinese medicine/ (10161)
60 exp oriental medicine/ (136)
61 exp herbaceous agent/ (13268)
62 exp medicinal plant/ (55133)
63 chinese medicine.mp. (12463)
64 chinese traditional medicine.mp. (383)
65 traditional chinese medicine.mp. (4641)
66 oriental medicine.mp. (470)
67 traditional oriental medicine.mp. (89)
68 oriental traditional medicine.mp. (21)
69 chinese herb$.mp. (4719)
70 chinese medicinal.mp. (1055)
71 medicinal plant$.mp. (23071)
72 herb$.mp. (50176)
73 (She Xiang Bao Xin or Su Xiao Jiu Xin or Fu Fang Dan Shen or Guan Xin Dan Shen or Su Bing or Su Xin or Tong Xin Luo or Hu Xin or Jiu Xin or Huan Xin or She Xiang Su He or She Xiang or Zhu She Xiao Shuan or Huo Xin Dan or Yi Xin Wan or Xin Ling Wan or She Xiang Xin Nao Le or Kuan Xiong or Xin Tong or Fu Fang Xi Xin or Guan Xin).mp. (23)
74 (Xin Jiao Tong Ning or Jiu Xin or Ge Gen Su or Shen Fu or Di Long or Hong Hua or Shu Xue Ning or Chuan Qiong Chin or Shu Xue Tong or Mai Pu Lin or Deng Zhan Xi Xin or Huang Qi or Ci Wu Jia or Guan Xin Ning or Tong Xin Luo or Nao Xin Tong or Bu Xin qi or Shen Shao or Nuo Di Kang or Yang Xin Shi or Xue Fu Zhu Yu or Xin Ke Shu).mp. (97)

75 (Di Ao Xin Xue Kang or Xin Yuan or Wen Xin or Sheng Mai or Yi Xin Shu or Xin Nao Shu Tong or Yin Xing Ye or Huo Xue Tong Mai or Xin Ke Ning or Xue Sai Tong or Xin Tong or Dan Lou or Xin Bao or You Gui or Xin Mai Tong or Tong Guan or Shen Mai or Deng Zhan Hua).mp. (50)
76 59 or 60 or 61 or 62 or 63 or 64 or 65 or 66 or 67 or 68 or 69 or 70 or 71 or 72 or 73 or 74 or 75 (99567)
77 39 and 58 and 76 (1487)
78 limit 77 to human (1321)
79 limit 78 to "review" (595)
80 78 not 79 (726)

4: AMED: Search strategy and results

1 Controlled study/ or Randomized Controlled Trial/ (1381)
2 Placebo/ (506)
3 Random$.tw. (11231)
4 latin square.tw. (24)
5 crossover.tw. (514)
6 cross-over.tw. (219)
7 placebo$.tw. (2345)
8 ((doubl$ or singl$ or tripl$ or trebl$) adj5 (blind$ or mask$)).tw. (1995)
9 (comparativ$ adj5 trial$).tw. (139)
10 (clinical adj5 trial$).tw. (4166)
11 exp traditional medicine chinese/ (4744)
12 exp drugs chinese herbal/ (1348)
13 exp plants medicinal/ (16139)
14 exp herbal drugs/ (6618)
15 herb$.mp. (10453)
16 (chinese adj5 medicine).mp. (5058)
17 (traditional chinese adj5 medicine).mp. (878)
18 (chinese adj5 drug$).mp. (1538)
19 (chinese adj5 herb$).mp. (1780)
20 (medic$ adj5 plant$).mp. (15307)
21 (herb$ adj5 plant$).mp. (442)
22 (She Xiang Bao Xin or Su Xiao Jiu Xin or Fu Fang Dan Shen or Guan Xin Dan Shen or Su Bing or Su Xin or Tong Xin Luo or Hu Xin or Jiu Xin or Huan Xin or She Xiang Su He or She Xiang or Zhu She Xiao Shuan or Huo Xin Dan or Yi Xin Wan or Xin Ling Wan or She Xiang Xin Nao Le or Kuan Xiong or Xin Tong or Fu Fang Xi Xin or Guan Xin).mp. (3)
23 (Xin Jiao Tong Ning or Jiu Xin or Ge Gen Su or Shen Fu or Di Long or Hong Hua or Shu Xue Ning or Chuan Qiong Chin or Shu Xue Tong or Mai Pu Lin or Deng Zhan Xi Xin or Huang Qi or Ci Wu Jia or Guan Xin Ning or Tong Xin Luo or Nao Xin Tong or Bu Xin qi or Shen Shao or Nuo Di Kang or Yang Xin Shi or Xue Fu Zhu Yu or Xin Ke Shu).mp. (11)
24 (Di Ao Xin Xue Kang or Xin Yuan or Wen Xin or Sheng Mai or Yi Xin Shu or Xin Nao Shu Tong or Yin Xing Ye or Huo Xue Tong Mai or Xin Ke Ning or Xue Sai Tong or Xin Tong or Dan Lou or Xin Bao or You Gui or Xin Mai Tong or Tong Guan or Shen Mai or Deng Zhan Hua).mp. (9)
25 (coronary adj3 disease$).mp. (922)
26 angina.mp. (241)
27 myocardial infarct$.mp. (586)
28 heart attack$.mp. (52)
29 (cardiovascular adj2 (disease$ or event$)).mp. (1300)
30 exp cardiovascular disease/ (11782)
31 25 or 26 or 27 or 28 or 29 or 30 (12285)

32 11 or 12 or 13 or 14 or 15 or 16 or 17 or 18 or 19 or 20 or 21 or 22 or 23 or 24 (26067)

33 1 or 2 or 3 or 4 or 5 or 6 or 7 or 8 or 9 or 10 or 11 or 12 or 13 or 14 or 15 or 16 or 17 or 18 or 19 or 20 or 21 or 22 or 23 or 24 (39508)

34 31 and 32 and 33 (946)

35 limit 34 to "review" (11)

36 34 not 35 (935)

5: CINAHL PLUS: search strategy and results

S30	S27 and S28	Limiters - Clinical Queries: Therapy - High Sensitivity; Human<br>Search modes - Boolean/Phrase	Interface - EBSCOhost<br>Search Screen - Advanced Search<br>Database - CINAHL Plus	1618
S29	S27 and S28	Search modes - Boolean/Phrase	Interface - EBSCOhost<br>Search Screen - Advanced Search<br>Database - CINAHL Plus	4726
S28	S14 or S15 or S16 or S17 or S18 or S19 or S20 or S21 or S22 or S23 or S24 or S25	Search modes - Boolean/Phrase	Interface - EBSCOhost<br>Search Screen - Advanced Search<br>Database - CINAHL Plus	Display
S27	S13 or S26	Search modes - Boolean/Phrase	Interface - EBSCOhost<br>Search Screen - Advanced Search<br>Database - CINAHL Plus	Display
S26	("cardiovascular") or (MH "Cardiovascular Abnormalities+") or (MH "Cardiovascular Diseases+") or (MH "Cardiovascular System+") or (MH "Surgery, Cardiovascular+") or (MH "Technology, Cardiovascular") or (MH "Cardiovascular Care") or (MH "Cardiovascular Nursing+")	Search modes - Boolean/Phrase	Interface - EBSCOhost<br>Search Screen - Advanced Search<br>Database - CINAHL Plus	Display
S25	plant extract$	Search modes - Boolean/Phrase	Interface - EBSCOhost<br>Search Screen - Advanced Search<br>Database - CINAHL Plus	Display
S24	Complementary Therap$	Search modes - Boolean/Phrase	Interface - EBSCOhost<br>Search Screen - Advanced	Display

			Search<br>Database - CINAHL Plus	
S23	alternative medicine	Search modes - Boolean/Phrase	Interface - EBSCOhost<br>Search Screen - Advanced Search<br>Database - CINAHL Plus	Display
S22	(MH "Medicine, Chinese Traditional+") or (MH "Medicine, Oriental Traditional+") or (MH "Medicine, Traditional+") or (MH "Traditional Healers")	Search modes - Boolean/Phrase	Interface - EBSCOhost<br>Search Screen - Advanced Search<br>Database - CINAHL Plus	Display
S21	herb	Search modes - Boolean/Phrase	Interface - EBSCOhost<br>Search Screen - Advanced Search<br>Database - CINAHL Plus	Display
S20	Medicinal Plant/	Search modes - Boolean/Phrase	Interface - EBSCOhost<br>Search Screen - Advanced Search<br>Database - CINAHL Plus	Display
S19	Chinese Herb/	Search modes - Boolean/Phrase	Interface - EBSCOhost<br>Search Screen - Advanced Search<br>Database - CINAHL Plus	Display
S18	Chinese Drug/	Search modes - Boolean/Phrase	Interface - EBSCOhost<br>Search Screen - Advanced Search<br>Database - CINAHL Plus	Display
S17	Plant Extract/	Search modes - Boolean/Phrase	Interface - EBSCOhost<br>Search Screen - Advanced Search<br>Database - CINAHL Plus	Display
S16	chinese medicine/	Search modes - Boolean/Phrase	Interface - EBSCOhost<br>Search Screen - Advanced Search<br>Database - CINAHL Plus	Display
S15	traditional medicine	Search modes - Boolean/Phrase	Interface - EBSCOhost<br>Search Screen - Advanced	Display

			Search<br>Database - CINAHL Plus	
S14	alternative medicine/	Search modes - Boolean/Phrase	Interface - EBSCOhost<br>Search Screen - Advanced Search<br>Database - CINAHL Plus	Display
S13	S1 or S2 or S3 or S4 or S5 or S6 or S7 or S8 or S9 or S10 or S11 or S12	Search modes - Boolean/Phrase	Interface - EBSCOhost<br>Search Screen - Advanced Search<br>Database - CINAHL Plus	Display
S12	(coronary n3 bypass$)	Search modes - Boolean/Phrase	Interface - EBSCOhost<br>Search Screen - Advanced Search<br>Database - CINAHL Plus	Display
S11	(heart n3 disease$)	Search modes - Boolean/Phrase	Interface - EBSCOhost<br>Search Screen - Advanced Search<br>Database - CINAHL Plus	Display
S10	(myocardial n3 infarct$)	Search modes - Boolean/Phrase	Interface - EBSCOhost<br>Search Screen - Advanced Search<br>Database - CINAHL Plus	Display
S9	(heart n3 infarct$)	Search modes - Boolean/Phrase	Interface - EBSCOhost<br>Search Screen - Advanced Search<br>Database - CINAHL Plus	Display
S8	angina	Search modes - Boolean/Phrase	Interface - EBSCOhost<br>Search Screen - Advanced Search<br>Database - CINAHL Plus	Display
S7	(coronary n3 disease$)	Search modes - Boolean/Phrase	Interface - EBSCOhost<br>Search Screen - Advanced Search<br>Database - CINAHL Plus	Display
S6	MH heart function test	Search modes - SmartText Searching	Interface - EBSCOhost<br>Search Screen - Advanced Search<br>Database - CINAHL Plus	Display
S5	Heart Disease/	Search modes - Boolean/Phrase	Interface - EBSCOhost<br>Search Screen - Advanced	Display

			Search<br>Database - CINAHL Plus	
S4	MH Ischemic Heart Disease	Search modes - SmartText Searching	Interface - EBSCOhost<br>Search Screen - Advanced Search<br>Database - CINAHL Plus	Display
S3	Heart Disease	Search modes - Boolean/Phrase	Interface - EBSCOhost<br>Search Screen - Advanced Search<br>Database - CINAHL Plus	Display
S2	MH heart surgery+	Search modes - Boolean/Phrase	Interface - EBSCOhost<br>Search Screen - Advanced Search<br>Database - CINAHL Plus	Display
S1	Cardiovascular Disease/	Search modes - Boolean/Phrase	Interface - EBSCOhost<br>Search Screen - Advanced Search<br>Database - CINAHL Plus	Display

6: CBM (China Biological Medicine Database, 中国生物医学文献数据库): search strategy and results

	Search Strategy	Results
1	主题词:临床试验/全部树/全部副主题词	110837
2	主题词:随机分配/全部树/全部副主题词	29819
3	主题词:双盲法/全部树/全部副主题词	3683
4	主题词:单盲法/全部树/全部副主题词	413
5	主题词:安慰剂/全部树/全部副主题词	2599
6	#5 or #4 or #3 or #2 or #1	143021
7	主题词=治疗应用	2084
8	#7 or #6	145039
9	中文标题:随机对照 or 临床试验 or 临床研究 or 临床观察 or 临床效果 or 临床分析 or 临床疗效 or 临床比较 or 对照研究 or 对照试验 or 对照治疗 or 对照观察 or 对照分析 or 对比研究 or 对比观察 or 对比分析 or 分组研究 or 比较研究 or 多中心研究 or 疗效观察 or 疗效评价 or 疗效分析 or 疗效比较 or 治疗研究 or 治疗比较 or 效果比较	457344
10	#9 or #8	559316
11	主题词:心肌缺血/全部树/ZD/ZJ	7128
12	主题词:中草药△/全部树/AE/PO/TU	54223
13	中文标题:丹参 or 葛根 or 细辛 or 红花 or 三七 or 刺五加 or 毛冬青 or 益母草 or 延胡索 or 赤芍 or 蒲黄 or 淫羊藿 or 瓜蒌 or 防己 or 灵芝 or 山楂 or 当归 or 川穹 or 麝香 or 苏合香 or 灯盏花 or 银杏叶 or 阿槐酸钠 or 速效救心 or 冠心苏合 or 苏心丸 or 益心丸 or 心灵丸 or 心宝 or 右归 or 护心丹 or 救心丹 or 环心丹 or 活心丹 or 苏冰滴丸 or 冠心膏 or 心绞痛宁膏 or 救心油 or 养心氏片 or 心可舒 or 稳心颗粒 or 丹七片 or 丹蒌片 or 心血宁 or 通心络 or 心脉通 or 脑心通 or 益心舒 or 心可宁 or 诺迪康 or 血脉康 or 心血康 or 珠麝消栓 or 心脑舒通 or 血府逐瘀 or 心元胶囊 or 通冠胶囊 or 活血通脉胶囊 or 补心气口服液 or 心通口服液 or 舒心口服液 or 滋心阴口服液 or 参芍 or 生脉 or 血塞通 or 血栓通 or 脉络宁 or 舒血宁 or 疏血通 or 冠心宁 or 脉普林 or 醒脑静 or 清开灵 or 参附注射液 or 参麦注射液 or 地龙注射液 or 黄芪注射液 or 宽胸气雾剂 or 心痛气雾剂	67281
14	关键词:丹参 or 葛根 or 细辛 or 红花 or 三七 or 刺五加 or 毛冬青 or 益母草 or 延胡索 or 赤芍 or 蒲黄 or 淫羊藿 or 瓜蒌 or 防己 or 灵芝 or 山楂 or 当归 or	42506

	Search Strategy	Results
	川穹 or 麝香 or 苏合香 or 灯盏花 or 银杏叶 or 阿槐酸钠 or 速效救心 or 冠心苏合 or 苏心丸 or 益心丸 or 心灵丸 or 心宝 or 右归 or 护心丹 or 救心丹 or 环心丹 or 活心丹 or 苏冰滴丸 or 冠心膏 or 心绞痛宁膏 or 救心油 or 养心氏片 or 心可舒 or 稳心颗粒 or 丹七片 or 丹蒌片 or 心血宁 or 通心络 or 心脉通 or 脑心通 or 益心舒 or 心可宁 or 诺迪康 or 血脉康 or 心血康 or 珠麝消栓 or 心脑舒通 or 血府逐瘀 or 心元胶囊 or 通冠胶囊 or 活血通脉胶囊 or 补心气口服液 or 心通口服液 or 舒心口服液 or 滋心阴口服液 or 参芍 or 生脉 or 血塞通 or 血栓通 or 脉络宁 or 舒血宁 or 疏血通 or 冠心宁 or 脉普林 or 醒脑静 or 清开灵 or 参附注射液 or 参麦注射液 or 地龙注射液 or 黄芪注射液 or 宽胸气雾剂 or 心痛气雾剂	
15	主题词:丹参 or 葛根 or 细辛 or 红花 or 三七 or 刺五加 or 毛冬青 or 益母草 or 延胡索 or 赤芍 or 蒲黄 or 淫羊藿 or 瓜蒌 or 防己 or 灵芝 or 山楂 or 当归 or 川穹 or 麝香 or 苏合香 or 灯盏花 or 银杏叶 or 阿槐酸钠 or 速效救心 or 冠心苏合 or 苏心丸 or 益心丸 or 心灵丸 or 心宝 or 右归 or 护心丹 or 救心丹 or 环心丹 or 活心丹 or 苏冰滴丸 or 冠心膏 or 心绞痛宁膏 or 救心油 or 养心氏片 or 心可舒 or 稳心颗粒 or 丹七片 or 丹蒌片 or 心血宁 or 通心络 or 心脉通 or 脑心通 or 益心舒 or 心可宁 or 诺迪康 or 血脉康 or 心血康 or 珠麝消栓 or 心脑舒通 or 血府逐瘀 or 心元胶囊 or 通冠胶囊 or 活血通脉胶囊 or 补心气口服液 or 心通口服液 or 舒心口服液 or 滋心阴口服液 or 参芍 or 生脉 or 血塞通 or 血栓通 or 脉络宁 or 舒血宁 or 疏血通 or 冠心宁 or 脉普林 or 醒脑静 or 清开灵 or 参附注射液 or 参麦注射液 or 地龙注射液 or 黄芪注射液 or 宽胸气雾剂 or 心痛气雾剂	67857
16	#13 or #14 or #15	87956
17	#16 or #12	128615
18	主题词:心肌缺血/全部树/全部副主题词	134169
19	#17 and #18	9745
20	#19 or #11	13049
21	#20 and #10	4772
22	#20 and #10 -限定:人类	4523
23	#20 and #10 -限定:综述	29
24	#20 and #10 -限定:讲座	0
25	#20 and #10 -限定:译文	0
26	#20 and #10 -限定:病例报告	5
27	#20 and #10 -限定:Meta分析	13
28	#27 or #26 or #25 or #24 or #23	47
29	(#22) not (#28)	4482

	Search Strategy	Results
30	中文标题:浅谈 or 浅析 or 初探 or 新用 or 巧用 or 漫谈 or 治验 or 体会 or 举验 or 举隅 or 举要 or 案例 or 刍议 or 经验 or 验案 or 配伍分析 or 概述	335093
31	(#29) not (#30)	4473
32	(#29) not (#30) -限定:动物	13
33	(#31) not (#32)	4460

7: CMCC (Chinese Medical Current Contents, 中文生物医学期刊文献数据库): search strategy and results

由于CMCC检索能力的限制，用六条表达式分别检索，然后将每条检索式的结果分别导出到文献管理软件，最后综合。六条表达式的结果一共为2279条。

#1 结果：16条

((临床试验/FLD=关键词 OR 对照试验/FLD=关键词 OR 随机/FLD=关键词) AND (冠心病/FLD=题名,关键词 OR 心肌梗死/FLD=题名,关键词 OR 心肌梗塞/FLD=题名,关键词 OR 心绞痛/FLD=题名,关键词) AND (丹参/FLD=题名,关键词 OR 葛根/FLD=题名,关键词 OR 细辛/FLD=题名,关键词 OR 红花/FLD=题名,关键词 OR 三七/FLD=题名,关键词 OR 刺五加/FLD=题名,关键词 OR 毛冬青/FLD=题名,关键词 OR 益母草/FLD=题名,关键词 OR 延胡索/FLD=题名,关键词 OR 赤芍/FLD=题名,关键词 OR 蒲黄/FLD=题名,关键词 OR 淫羊藿/FLD=题名,关键词 OR 瓜蒌/FLD=题名,关键词 OR 防己/FLD=题名,关键词 OR 灵芝/FLD=题名,关键词 OR 山楂/FLD=题名,关键词 OR 当归/FLD=题名,关键词 OR 川穹/FLD=题名,关键词 OR 麝香/FLD=题名,关键词 OR 苏合香/FLD=题名,关键词 OR 灯盏花/FLD=题名,关键词 OR 银杏叶/FLD=题名,关键词 OR 阿槐酸钠/FLD=题名,关键词 OR 速效救心/FLD=题名,关键词 OR 冠心苏合/FLD=题名,关键词 OR 苏心丸/FLD=题名,关键词 OR 益心丸/FLD=题名,关键词 OR 心灵丸/FLD=题名,关键词 OR 心宝/FLD=题名,关键词 OR 右归/FLD=题名,关键词 OR 护心丹/FLD=题名,关键词 OR 救心丹/FLD=题名,关键词 OR 环心丹/FLD=题名,关键词 OR 活心丹/FLD=题名,关键词 OR 苏冰滴丸/FLD=题名,关键词 OR 冠心膏/FLD=题名,关键词)) SUB (#文献类型=Z OR #文献类型=J OR #文献类型=W OR #文献类型=Y OR 动物/FLD=关键词 OR 浅谈/FLD=题名 OR 浅析/FLD=题名 OR 初探/FLD=题名 OR 新用/FLD=题名 OR 巧用/FLD=题名 OR 漫谈/FLD=题名 OR 治验/FLD=题名 OR 体会/FLD=题名 OR 举验/FLD=题名 OR 举隅/FLD=题名 OR 举要/FLD=题名 OR 案例/FLD=题名 OR 刍议/FLD=题名 OR 经验/FLD=题名 OR 验案/FLD=题名 OR 配伍分析/FLD=题名 OR 概述/FLD=题名)

#2 结果：926条

((随机/FLD=摘要 AND 对照/FLD=摘要) AND (冠心病/FLD=题名,关键词 OR 心肌梗死/FLD=题名,关键词 OR 心肌梗塞/FLD=题名,关键词 OR 心绞痛/FLD=题名,关键词) AND (丹参/FLD=题名,关键词 OR 葛根/FLD=题名,关键词 OR 细辛/FLD=题名,关键词 OR 红花/FLD=题名,关键词 OR 三七/FLD=题名,关键词 OR 刺五加/FLD=题名,关键词 OR 毛冬青/FLD=题名,关键词 OR 益母草/FLD=题名,关键词 OR 延胡索/FLD=题名,关键词 OR 赤芍/FLD=题名,关键词 OR 蒲黄/FLD=题名,关键词 OR 淫羊藿/FLD=题名,关键词 OR 瓜蒌/FLD=题名,关键词 OR 防己/FLD=题名,关键词 OR 灵芝/FLD=题名,关键词 OR 山楂/FLD=题名,关键词 OR 当归/FLD=题名,关键词 OR 川穹/FLD=题名,关键词 OR 麝香/FLD=题名,关键词 OR 苏合香/FLD=题名,关键词 OR 灯盏花/FLD=题名,关键词 OR 银杏

叶/FLD=题名,关键词 OR 阿槐酸钠/FLD=题名,关键词 OR 速效救心/FLD=题名,关键词 OR 冠心苏合/FLD=题名,关键词 OR 苏心丸/FLD=题名,关键词 OR 益心丸/FLD=题名,关键词 OR 心灵丸/FLD=题名,关键词 OR 心宝/FLD=题名,关键词 OR 右归/FLD=题名,关键词 OR 护心丹/FLD=题名,关键词 OR 救心丹/FLD=题名,关键词 OR 环心丹/FLD=题名,关键词 OR 活心丹/FLD=题名,关键词 OR 苏冰滴丸/FLD=题名,关键词 OR 冠心膏/FLD=题名,关键词)) SUB (#文献类型=Z OR #文献类型=J OR #文献类型=W OR #文献类型=Y OR 动物/FLD=关键词 OR 浅谈/FLD=题名 OR 浅析/FLD=题名 OR 初探/FLD=题名 OR 新用/FLD=题名 OR 巧用/FLD=题名 OR 漫谈/FLD=题名 OR 治验/FLD=题名 OR 体会/FLD=题名 OR 举验/FLD=题名 OR 举隅/FLD=题名 OR 举要/FLD=题名 OR 案例/FLD=题名 OR 刍议/FLD=题名 OR 经验/FLD=题名 OR 验案/FLD=题名 OR 配伍分析/FLD=题名 OR 概述/FLD=题名)

#3 结果：6条

((临床试验/FLD=关键词 OR 对照试验/FLD=关键词 OR 随机/FLD=关键词) AND (冠心病/FLD=题名,关键词 OR 心肌梗死/FLD=题名,关键词 OR 心肌梗塞/FLD=题名,关键词 OR 心绞痛/FLD=题名,关键词) AND (心绞痛宁膏/FLD=题名,关键词 OR 救心油/FLD=题名,关键词 OR 养心氏片/FLD=题名,关键词 OR 心可舒/FLD=题名,关键词 OR 稳心颗粒/FLD=题名,关键词 OR 丹七片/FLD=题名,关键词 OR 丹蒌片/FLD=题名,关键词 OR 心血宁/FLD=题名,关键词 OR 通心络/FLD=题名,关键词 OR 心脉通/FLD=题名,关键词 OR 脑心通/FLD=题名,关键词 OR 益心舒/FLD=题名,关键词 OR 心可宁/FLD=题名,关键词 OR 诺迪康/FLD=题名,关键词 OR 血脉康/FLD=题名,关键词 OR 心血康/FLD=题名,关键词 OR 珠麝消栓/FLD=题名,关键词 OR 心脑舒通/FLD=题名,关键词 OR 血府逐瘀/FLD=题名,关键词 OR 心元胶囊/FLD=题名,关键词 OR 通冠胶囊/FLD=题名,关键词 OR 活血通脉胶囊/FLD=题名,关键词 OR 补心气口服液/FLD=题名,关键词 OR 心通口服液/FLD=题名,关键词 OR 舒心口服液/FLD=题名,关键词 OR 滋心阴口服液/FLD=题名,关键词 OR 参芍/FLD=题名,关键词 OR 生脉/FLD=题名,关键词 OR 血塞通/FLD=题名,关键词 OR 血栓通/FLD=题名,关键词 OR 脉络宁/FLD=题名,关键词 OR 舒血宁/FLD=题名,关键词 OR 疏血通/FLD=题名,关键词 OR 冠心宁/FLD=题名,关键词 OR 脉普林/FLD=题名,关键词 OR 醒脑静/FLD=题名,关键词 OR 清开灵/FLD=题名,关键词 OR 参附注射液/FLD=题名,关键词 OR 参麦注射液/FLD=题名,关键词 OR 地龙注射液/FLD=题名,关键词 OR 黄芪注射液/FLD=题名,关键词 OR 宽胸气雾剂/FLD=题名,关键词 OR 心痛气雾剂/FLD=题名,关键词)) SUB (#文献类型=Z OR #文献类型=J OR #文献类型=W OR #文献类型=Y OR 动物/FLD=关键词 OR 浅谈/FLD=题名 OR 浅析/FLD=题名 OR 初探/FLD=题名 OR 新用/FLD=题名 OR 巧用/FLD=题名 OR 漫谈/FLD=题名 OR 治验/FLD=题名 OR 体会/FLD=题名 OR 举验/FLD=题名 OR 举隅/FLD=题名 OR 举要/FLD=题名 OR 案例/FLD=题名 OR 刍议/FLD=题名 OR 经验/FLD=题名 OR 验案/FLD=题名 OR 配伍分析/FLD=题名 OR 概述/FLD=题名)

#4 结果：981条

((随机/FLD=摘要 AND 对照/FLD=摘要) AND (冠心病/FLD=题名,关键词 OR 心肌梗死/FLD=题名,关键词 OR 心肌梗塞/FLD=题名,关键词 OR 心绞痛/FLD=题名,

关键词) AND (心绞痛宁膏/FLD=题名,关键词 OR 救心油/FLD=题名,关键词 OR 养心氏片/FLD=题名,关键词 OR 心可舒/FLD=题名,关键词 OR 稳心颗粒/FLD=题名,关键词 OR 丹七片/FLD=题名,关键词 OR 丹蒌片/FLD=题名,关键词 OR 心血宁/FLD=题名,关键词 OR 通心络/FLD=题名,关键词 OR 心脉通/FLD=题名,关键词 OR 脑心通/FLD=题名,关键词 OR 益心舒/FLD=题名,关键词 OR 心可宁/FLD=题名,关键词 OR 诺迪康/FLD=题名,关键词 OR 血脉康/FLD=题名,关键词 OR 心血康/FLD=题名,关键词 OR 珠麝消栓/FLD=题名,关键词 OR 心脑舒通/FLD=题名,关键词 OR 血府逐瘀/FLD=题名,关键词 OR 心元胶囊/FLD=题名,关键词 OR 通冠胶囊/FLD=题名,关键词 OR 活血通脉胶囊/FLD=题名,关键词 OR 补心气口服液/FLD=题名,关键词 OR 心通口服液/FLD=题名,关键词 OR 舒心口服液/FLD=题名,关键词 OR 滋心阴口服液/FLD=题名,关键词 OR 参芎/FLD=题名,关键词 OR 生脉/FLD=题名,关键词 OR 血塞通/FLD=题名,关键词 OR 血栓通/FLD=题名,关键词 OR 脉络宁/FLD=题名,关键词 OR 舒血宁/FLD=题名,关键词 OR 疏血通/FLD=题名,关键词 OR 冠心宁/FLD=题名,关键词 OR 脉普林/FLD=题名,关键词 OR 醒脑静/FLD=题名,关键词 OR 清开灵/FLD=题名,关键词 OR 参附注射液/FLD=题名,关键词 OR 参麦注射液/FLD=题名,关键词 OR 地龙注射液/FLD=题名,关键词 OR 黄芪注射液/FLD=题名,关键词 OR 宽胸气雾剂/FLD=题名,关键词 OR 心痛气雾剂/FLD=题名,关键词 OR 补心气口服液/FLD=题名,关键词 OR 心通口服液/FLD=题名,关键词 OR 舒心口服液/FLD=题名,关键词 OR 滋心阴口服液/FLD=题名,关键词 OR 参芎/FLD=题名,关键词 OR 生脉/FLD=题名,关键词 OR 血塞通/FLD=题名,关键词 OR 血栓通/FLD=题名,关键词 OR 脉络宁/FLD=题名,关键词 OR 舒血宁/FLD=题名,关键词 OR 疏血通/FLD=题名,关键词 OR 冠心宁/FLD=题名,关键词 OR 脉普林/FLD=题名,关键词 OR 醒脑静/FLD=题名,关键词 OR 清开灵/FLD=题名,关键词 OR 参附注射液/FLD=题名,关键词 OR 参麦注射液/FLD=题名,关键词 OR 地龙注射液/FLD=题名,关键词 OR 黄芪注射液/FLD=题名,关键词 OR 宽胸气雾剂/FLD=题名,关键词 OR 心痛气雾剂/FLD=题名,关键词)) SUB (#文献类型=Z OR #文献类型=J OR #文献类型=W OR #文献类型=Y OR 动物/FLD=关键词 OR 浅谈/FLD=题名 OR 浅析/FLD=题名 OR 初探/FLD=题名 OR 新用/FLD=题名 OR 巧用/FLD=题名 OR 漫谈/FLD=题名 OR 治验/FLD=题名 OR 体会/FLD=题名 OR 举验/FLD=题名 OR 举隅/FLD=题名 OR 举要/FLD=题名 OR 案例/FLD=题名 OR 刍议/FLD=题名 OR 经验/FLD=题名 OR 验案/FLD=题名 OR 配伍分析/FLD=题名 OR 概述/FLD=题名)

#5 结果：9条

((临床试验/FLD=关键词 OR 对照试验/FLD=关键词 OR 随机/FLD=关键词) AND (冠心病/FLD=题名,关键词 OR 心肌梗死/FLD=题名,关键词 OR 心肌梗塞/FLD=题名,关键词 OR 心绞痛/FLD=题名,关键词) AND (中药/FLD=关键词 OR 中草药/FLD=关键词 OR 中成药/FLD=关键词 OR 中西医结合/FLD=关键词)) SUB (#文献类型=Z OR #文献类型=J OR #文献类型=W OR #文献类型=Y OR 动物/FLD=关键词 OR 浅谈/FLD=题名 OR 浅析/FLD=题名 OR 初探/FLD=题名 OR 新用/FLD=题名 OR 巧用/FLD=题名 OR 漫谈/FLD=题名 OR 治验/FLD=题名 OR 体会/FLD=题名 OR 举验/FLD=题名 OR 举隅/FLD=题名 OR 举要/FLD=题名 OR 案例/FLD=题名 OR 刍议/FLD=题名 OR 经验/FLD=题名 OR 验案/FLD=题名 OR 配伍分析/FLD=题名 OR 概述/FLD=题名)

#6 结果：341条

((随机/FLD=摘要 AND 对照/FLD=摘要) AND (冠心病/FLD=题名,关键词 OR 心肌梗死/FLD=题名,关键词 OR 心肌梗塞/FLD=题名,关键词 OR 心绞痛/FLD=题名,关键词) AND (中药/FLD=关键词 OR 中草药/FLD=关键词 OR 中成药/FLD=关键词 OR 中西医结合/FLD=关键词)) SUB (#文献类型=Z OR #文献类型=J OR #文献类型=W OR #文献类型=Y OR 动物/FLD=关键词 OR 浅谈/FLD=题名 OR 浅析/FLD=题名 OR 初探/FLD=题名 OR 新用/FLD=题名 OR 巧用/FLD=题名 OR 漫谈/FLD=题名 OR 治验/FLD=题名 OR 体会/FLD=题名 OR 举验/FLD=题名 OR 举隅/FLD=题名 OR 举要/FLD=题名 OR 案例/FLD=题名 OR 刍议/FLD=题名 OR 经验/FLD=题名 OR 验案/FLD=题名 OR 配伍分析/FLD=题名 OR 概述/FLD=题名)

8: TCMLARS (Traditional Chinese Medical Literature Analysis and Retrieval System, 中国中医药期刊文献数据库): search strategy and results

结果为4117条

检索表达式：

((((WXMES=心肌梗塞V中医药疗法) OR (WXMES=心肌梗塞V中西医结合疗法)) OR (WXMES = ('心肌顿抑V中医药疗法','心肌顿抑V中西医结合疗法','休克，心原性V中医药疗法','休克，心原性V中西医结合疗法','心肌顿抑V中医药疗法','心肌顿抑V中西医结合疗法','无复流现象V中医药疗法','无复流现象V中西医结合疗法','休克，心原性V中医药疗法','休克，心原性V中西医结合疗法'))) OR (((WXMES=心绞痛V中医药疗法) OR (WXMES=心绞痛V中西医结合疗法)) OR (WXMES = ('心绞痛，不稳定型V中医药疗法','心绞痛，不稳定型V中西医结合疗法','心绞痛，变异型V中医药疗法','心绞痛，变异型V中西医结合疗法','微血管性心绞痛V中医药疗法','微血管性心绞痛V中西医结合疗法','心绞痛，不稳定型V中医药疗法','心绞痛，不稳定型V中西医结合疗法','心绞痛，变异型V中医药疗法','心绞痛，变异型V中西医结合疗法','微血管性心绞痛V中医药疗法','微血管性心绞痛V中西医结合疗法','心绞痛，不稳定型V中医药疗法','心绞痛，不稳定型V中西医结合疗法','心绞痛，变异型V中医药疗法','心绞痛，变异型V中西医结合疗法'))) AND (WXTYP=随机对照试验 OR WXTAG=随机对照试验 OR WXMES=随机对照试验) AND (WXTAG=人类 OR WXTYP=人类 OR WXMES=人类) NOT (WXTAG=动物 OR WXTYP=动物 OR WXMES=动物)) NOT (WXTIC=浅谈 or 浅析 or 初探 or 新用 or 巧用 or 漫谈 or 治验 or 体会 or 举验 or 举隅 or 举要 or 案例 or 刍议 or 经验 or 验案 or 配伍分析 or 概述)

Apendix2 Searching strategy of systematic reviews on traditional Chinese medicine in the Cochrane Database of Systematic Reviews (CDSR)

#1 MeSH descriptor: [Medicine, East Asian Traditional] explode all trees 762
#2 MeSH descriptor: [Phytotherapy] explode all trees 2928
#3 Enter terms for search: Medicine, Chinese Traditional:ti,ab 1612
#4 Enter terms for search: Plant*:ti,ab 2306
#5 Enter terms for search: chinese herb*:ti,ab 1197
#6 Enter terms for search: chinese adj (traditional or medicine*):ti,ab 72
#7 Enter terms for search: Herb*:ti,ab 2266
#8 Enter terms for search: traditional chinese med*:ti,ab 2888
#9 MeSH descriptor: [Medicine, Chinese Traditional] explode all trees 705
#10 MeSH descriptor: [Drugs, Chinese Herbal] explode all trees 2226
#11 MeSH descriptor: [acupuncture therapy] explode all trees 2688
#12 MeSH descriptor: [electroacupuncture] explode all trees 415
#13 MeSH descriptor: [acupunct* or electroacupunct* or electro-acupunct*] explode all trees 7010
#14 Enter terms for search#1 or #2 or #3 or #4 or #5 or #6 or #7 or #8 or #9 or #10 or #11 or #12 or #13 625 in Cochrane Reviews (Reviews only) 625
